# Glances at the Hospitals

**Published:** 1901-10-19

**Authors:** 


### GLANCES AT THE HOSPITALS.

### BRISTOL GENERAL HOSPITAL.

The total ordinary revenue was £10,586, that being £1,505 less than it was in the previous year, and the total ordinary expenditure was £11,291. The balance due to the treasurer increased from £357 to £2,163. It would seem that the subscriptions, instead of increasing, are actually less than they were ten years ago, but in this connection it should be stated that the workmen subscribed £1,927. Legacies to the amount of £5,500 have been received, but this sum cannot be invested, as £9,000 will be required to meet the expenses of various improvements now being carried out. At the end of the year there were 159 patients in the hospital, and 2,110 were admitted during it. Of these, 1,389 were discharged recovered, 405 relieved, 48 not improved, 68 at own request, one for misconduct, and 194 died. The average daily number of in-patients was 162. The total number of out-patients was 33,325. We looked in vain for a statement of the cost per bed occupied, or the cost per week, or the average stay in the hospital. The medical and surgical reports are hardly what might be expected in a hospital of this size and reputation, but we get an analysis of the most important medical and surgical cases, and this analysis shows the numbers cured, relieved, and died. There were 65 cases of enteric fever, of whom 11 died, and 46 cases of pneumonia, of whom 13 died. Of hernia 51 cases are mentioned, four of whom died; but what particular forms of hernia were under treatment, or what were the causes of death we hear nothing; and of aneurism there were two cases treated successfully, but we are not told which vessel was affected or the treatment employed. There is no operation table and no table of anæsthetics.

### RADCLIFFE INFIRMARY, OXFORD.

The ordinary receipts amounted to £6,919, and the ordinary expenditure to £7,992. The special expenditure was £452. To meet the deficit in part, stock to the value of £384 was sold, and £93 was transferred from the Establishment Fund, but in spite of this a debit balance of £2,617 had to be carried forward. The committee state that this deficit "compels them to repeat most urgently their appeal for more assistance;" but they add that the donations have enabled them to retain the capital not diminished. Every-one knows that a hospital should rely almost entirely on its annual income, and if donations be used as income and stock sold to meet current expenses, an end must speedily come to much of the usefulness of the institution. Forty-seven patients remained in the medical wards on the first day of the year, and 593 were admitted, making a total of 640 under treatment during the year. Of these 270 were discharged cured, 10 at own request, 39 not improved, 31 made out-patients, 179 relieved, 8 transferred, and 61 died. In the surgical wards there were 68 patients on January 1st, and 1,371 were admitted during the year. Of these 918 were cured, 283 relieved, 107 discharged for other reasons, 55 died, and 76 remained under treatment. In the out-patients' department there were 4,201 cases under treatment. The medical and surgical tables merely show the numbers under treatment for the various diseases, but a table of the principal operations is given, and this table shows the results. There were 26 amputations and excisions, and all these were successful. Of 72 operations for the radical cure of hernia, all except one were successful. An instructive table showing the cause of death in all the fatal cases during the year is printed on page 89, and we are pleased to note the presence of another table showing the nature of the anæsthetics administered during the surgical operations. A mixture of gas and ether was given 491 times, ether alone 429 times, chloroform 26 times, and a mixture of gas, ether and chloroform 29 times.

